# MDMA-assisted psychotherapy for people diagnosed with treatment-resistant PTSD: what it is and what it isn’t

**DOI:** 10.1186/s12991-020-00283-6

**Published:** 2020-05-12

**Authors:** Louise Morgan

**Affiliations:** Centre for Veterans’ Health, King Edward Vii’s Hospital, 5-10 Beaumont Street, Marylebone, London, W1G 6AA UK

**Keywords:** Posttraumatic stress disorder, PTSD, Treatment-resistant PTSD, MDMA, MDMA-assisted psychotherapy

## Abstract

**Background:**

PTSD is a chronic condition with high rates of comorbidity, but current treatment options are limited and not always effective. One novel approach is MDMA-assisted psychotherapy for people diagnosed with treatment-resistant PTSD, where MDMA is used as a catalyst to facilitate trauma processing during psychotherapy. The aim was to review all current research into MDMA-assisted psychotherapy for PTSD.

**Methods:**

Articles were identified through PubMed and Science Direct for items published up to 31st March 2019 using terms “treatments for PTSD”, “drug treatments for PTSD”, “MDMA”, “MDMA pathway”, “MDMA-assisted psychotherapy” and “MDMA-assisted psychotherapy for PTSD”. Articles were identified through Google Scholar and subject-specific websites. Articles and relevant references cited in those articles were reviewed.

**Results:**

Small-scale studies have shown reduced psychological trauma, however there has been widespread misunderstanding of the aims and implications of this work, most commonly the notion that MDMA is a ‘treatment for PTSD’, which to date has not been researched. This has harmful consequences, namely dangerous media reporting and impeding research progression in an already controversial field.

**Conclusions:**

MDMA-assisted psychotherapy may help people who have experienced psychological trauma and who have not been able to resolve their problems through existing treatments, however more research is needed. If this is to get appropriate research attention, we must report this accurately and objectively.

## Background

PTSD includes four symptom clusters that can develop after exposure to a traumatic event (DSM-5 [1]): re-experiencing, avoidance, negative alterations in cognition/mood and alterations in arousal and reactivity. People diagnosed with PTSD commonly avoid trauma-related thoughts and emotions and discussion of the traumatic event; however, the event is often relived through intrusive, recurrent recollections, dissociative episodes (flashbacks) and nightmares. There are no PTSD-specific drug treatments. UK NICE guidance [2] recommends antidepressants including venlafaxine or a selective serotonin reuptake inhibitor, such as sertraline, or an antipsychotic medication, such as risperidone, if there are signs of psychosis. These medications can alleviate certain feelings associated with a PTSD diagnosis, but show modest effects and do not target specifically any PTSD pathways, due to the fact that these biological pathways are not currently well understood [3, 4]. Trauma-focussed psychotherapies are therefore recommended as first-line treatments [5]. While these can be effective, drop-out rates are high (around 30% [6]), up to 58% of study participants still meet diagnostic criteria after treatment and only 32–66% reach a good level of functioning [7, 8].

The aim of this paper is to describe how MDMA might assist psychotherapy in the treatment of PTSD, to provide an overview of published research of MDMA-assisted psychotherapy and examples of misrepresentation of this in academic literature, and to demonstrate some of the negative consequences of misinterpretation of the research, to highlight why it is essential that we take a responsible approach to its reporting.

## Method

References for this review were identified through a search of PubMed and Science Direct for articles published up to March 2019 by the use of the terms “treatments for PTSD”, “drug treatments for PTSD”, “MDMA”, “MDMA pathway”, “MDMA mechanisms”, “MDMA-assisted psychotherapy” and “MDMA-assisted psychotherapy for PTSD”. Relevant articles published until March 2019 were also identified through Google Scholar and subject-specific websites (NICE: the National Institute for Health and Care Excellence and the Multidisciplinary Association for Psychedelic Studies). Articles resulting from these searches and relevant references cited in those articles were reviewed. All articles identified were in the English language. As this was a review of published research, approval from a research ethics committee was not required.

## Results and discussion

### The possible role of MDMA in assisting psychotherapy for PTSD

There are a number of possible reasons for limited effectiveness of current PTSD treatments. Psychotherapy is not a ‘quick fix’ and it can often require a long-term commitment to attend regular therapy sessions over a number of years. Additionally, some people who have experienced psychological trauma find it difficult to access buried traumatic memories and to then deal with the feelings that arise with their retrieval, causing them such distress that they are not able to continue with therapy [9]. Psychological trauma may also affect people’s ability to form trusting relationships, which impacts on the development of the vital partnership between therapist and client [10]. The cycle of avoidance and re-experiencing a traumatic event is thought to persist due to a deficit in the extinction of fear conditioning [11, 12] and under Foa and Kozak’s emotional processing theory [13], fear reduction can only be achieved when information incompatible with the fear structure is incorporated. Attending to threat is essential for this [14, 15], however this is inherently problematic for people diagnosed with PTSD.

MDMA is a monoamine releaser and promotes the release of serotonin, dopamine and noradrenaline [16, 17] and elevates serum oxytocin [18, 19] and brain imaging studies show reduced amygdala activity after MDMA administration [20]. The resulting effect is decreased hypervigilance and anxiety, increased relaxation and enhanced mood [21]. A combined treatment of MDMA and psychotherapy may be effective for treating PTSD as MDMA appears to diminish the fear response and decrease defensiveness without blocking access to memories [22], and may facilitate engagement in therapy by strengthening the therapeutic alliance and enhancing the identification of and response to emotional states [23]. Individuals are able to stay emotionally engaged with traumatic memories without being overwhelmed by anxiety or other painful emotions or avoiding them by dissociation or emotional numbing [24]. To be clear, it is not being suggested that MDMA-assisted psychotherapy is appropriate for everybody who is dealing with the consequences of psychological trauma. Most people can, over time, begin to build up a trusting relationship with a psychotherapist and start to think and talk about the traumatic event that they are trying to avoid, and then begin to work through their feelings. However, some people are not able to do this [13] and it is for these people that MDMA-assisted psychotherapy might be helpful. MDMA appears to increase feelings of trust, bring repressed, traumatic memories to the fore and reduce the fear normally associated with these memories [25].

### Clinical trials of MDMA-assisted psychotherapy for people diagnosed with treatment-resistant PTSD

The Multidisciplinary Association for Psychedelic Studies (MAPS) in the United States report a series of six phase 2 clinical trials looking at MDMA-assisted psychotherapy for PTSD (for a detailed summary see Feduccia et al. [26]). Participants met DSM-IV-R criteria for a diagnosis of PTSD and had treatment-resistant symptoms following at least 3 months of antidepressant treatment in addition to at least 6 months of psychotherapy. They were given preparatory, non-drug psychotherapy, two or three 8-h MDMA-assisted psychotherapy sessions and follow-up non-drug psychotherapy. The psychotherapy is detailed in the manual for MDMA-assisted psychotherapy [24]. Essentially the team use a nondirective approach that focuses on building a relationship between the participant and therapists to support the participant through their memories of traumatic events so they can reach a resolution and gain new perspectives about the meaning of these events. “The therapists’ responsibility is primarily to follow and facilitate rather than direct the experience” [24, p. 31]. It should be noted that a contract is made before the session that if the traumatic event does not come up spontaneously the therapist will bring it up, but the authors report that thus far the event has always been raised by the participant [24, p. 66].

These studies (combined *N* = 103) showed promising findings in terms of reduced psychological trauma and long-term remission (as assessed up to 6 years later) and with no drug-related serious adverse events and no adverse neurocognitive effects. 53% of participants in the active dose groups (75–125 mg of MDMA) group did not meet PTSD criteria on the Clinician Administered PTSD Scale-4 (CAPS-4) at the primary endpoint compared to 23% in the control groups (0–40 mg of MDMA) [27]. In collaboration with the US Food and Drug Administration (FDA) and the European Medicines Agency (EMA), this data supported the development of multi-site phase 3 clinical trials. This began in the US in 2018, and the European sites (the UK, Netherlands, Germany and the Czech Republic) are in the process of seeking approvals and are projected to start later in 2019. The phase 3 trials will compare the effects of three experimental sessions of MDMA-assisted psychotherapy versus placebo drug and psychotherapy. The treatments will take place during an approximately 12-week treatment period, which includes three drug-free preparatory sessions, three experimental (MDMA) sessions and nine drug-free integration sessions. Drug safety will be assessed by measuring blood pressure, heart rate and body temperature during experimental sessions, noting any adverse events and monitoring suicidal thoughts or behaviours. The trial aims to recruit up to 300 participants and if the findings are similar to those of the pilot studies, it is anticipated that the US FDA will approve MDMA-assisted psychotherapy as a recognised treatment for people diagnosed with treatment-resistant PTSD by 2021.

### Misinterpretation and its consequences

A review of the literature reveals much misunderstanding and misinterpretation of the MAPS research, its aims and what it tells us, and this has significant and potentially dangerous consequences. Research into the clinical use of illegal substances is highly controversial and misrepresentation of the research hampers progress in a field already fraught with legal and political challenges. MDMA was classed as a Schedule 1 drug in 1986 by the United Nations Convention on Psychotropic Substances of 1971 [28], meaning it is illegal and considered to be dangerous without therapeutic value, thus carrying out clinical research with MDMA is extremely difficult. Under the convention, the use of Schedule 1 substances is severely restricted. Countries who have signed the convention agree that any use is to be approved by those in medical or scientific establishments that are directly under the control of their Government. In the UK, control is exercised by the Home Office. Any clinical research with Schedule 1 drugs requires researchers, production sites and distributors to acquire a specific Home Office licence, which is expensive and time consuming, and undergo special criminal records checks and regular police inspections of laboratories (for an overview of the law and its impact on research, see Nutt et al. [29]).

The way the work has been (mis)understood has contributed to dangerous (mis)reporting by the media, potentially leading people to believe that MDMA will ‘cure’ them of psychological trauma. Examples include:

“MDMA ‘cures’ sufferers’ post-traumatic stress disorder in a few weeks during study: those on higher doses experience greater decreases in PTSD symptoms, finds pilot” (The Independent [30]).

“‘My therapist gave me a pill’: can MDMA help cure trauma? The ‘party drug’ is synonymous with rave culture, but an ambitious clinical study could prove it has the power to treat PTSD” (The Guardian [31]).

“Ecstasy could provide breakthrough therapy for soldiers suffering from PTSD, study finds. Traumatised combat veterans could soon be treated with MDMA after recreational drugs found to help those suffering from nervous conditions” (The Independent [32]).

### Aims and terminology

While we cannot control what the media writes, we can make sure we report research responsibly, and this starts with being clear about the aims of the work and the terminology used to describe it. The MAPS studies are unique in the world of clinical trials, in that they are a combination of psychotherapeutic intervention and a catalysing psychopharmacological treatment to facilitate trauma processing [33]. “MDMA is not just an augmenting add-on medication, but rather a catalyst that dramatically influences the psychotherapeutic process itself” [33, p. 50]. The MAPS authors are very clear to state in all their publications that it is the psychotherapy that is the treatment and that the MDMA facilitates the psychotherapy; “…the therapeutic effect is not due simply to the physiological effects of the medicine; rather, it is the result of an interaction between the effects of the medicine, the therapeutic setting and the mindsets of the participant and the therapists” [24, p. 6]. Despite this clarity, academic authors have made such statements as:

- “…multisite trials are necessary … to see MDMA become a licensed medicine. This phase of clinical MDMA research is now underway” [34, p. 2].
- “…putting MDMA on course to becoming a licensed treatment in 2021” [34, p.2].
- “Within 5 years, science will likely have answered a controversial question decades in the making: can the psychoactive drug commonly known as ecstasy…(MDMA) be used to treat psychiatric disorders?” [35, p. 419].
- “MDMA…was designated a breakthrough therapy for PTSD by the FDA” [36, p. 2].
- “…using…(MDMA) as a treatment for PTSD” [37, p. 176].
- None of these accurately reflect the MAPS work. Misrepresentation continues with the terminology used, that suggests that MDMA is a treatment for PTSD or that MDMA has a direct impact on PTSD, for example:
- “…(MDMA) as a treatment for PTSD” [37, p. 176].
- “MDMA therapy” [34, p. 4].
- “MDMA psychotherapy” [34, p. 2].
- “MDMA trials for PTSD” [34, p. 2].
- “MDMA-PTSD studies” [37, p. 179].
- “…the use of MDMA in PTSD” [38, p. 34].
- “…(MDMA’s) impact on posttraumatic stress disorder” [39, p. 908].
- “…the role of…(MDMA) on posttraumatic stress disorder” [39, p. 908].

The work should always be referred to as ‘MDMA-assisted psychotherapy’, or abbreviated to MDMA-AP [40]. MDMA has not been posited as a ‘treatment for PTSD’ and MAPS have not studied MDMA as a therapy; these are separate issues that, to date, nobody has researched.

There is additional misrepresentation of the role of MDMA in the therapeutic process, for example:

- “MDMA…as a…co-drug for therapy” [41, p. 114].
- “…MDMA associated to psychotherapy” [38, p. 433].
- “MDMA paired with psychological treatment” [42, p. 7].
- “MDMA…supplementary to…” [42, p. 7].
- “…MDMA enhancement…” [42, p. 8].
- “…MDMA as an adjunctive to treatment…” [42, p. 8].
- “…studies examined the augmentation effects of MDMA…” [42, p. 7].
- “…MDMA…as an adjunct to post-trauma psychological therapy” [43, p. 4].
- “…(MDMA)…could be a useful adjunct to psychotherapy” [37, p. 177].
- “…MDMA prescribed as an adjunct for treating PTSD” [37, p. 179].

MDMA has not been researched as a co-drug, it is not associated to or paired with psychotherapy, it is not supplementary to treatment and MDMA has not been used as an adjunct (“a thing added to something else as a supplementary rather than an essential part” [44]). Authors have even confused (sometimes within the same paper) whether they think MDMA has been researched as an adjunct to psychotherapy or as an adjunct for treating PTSD. It has not been researched as an adjunct to either.

Adding to the confusion is the interchangeable use of the terms ‘ecstasy’ and ‘MDMA’, when they are not the same [23]. Substances sold under the name ‘ecstasy’ may contain MDMA, but frequently also contain unknown and/or dangerous additional components. In the laboratory, pure MDMA has been proven sufficiently safe for human consumption when taken a limited number of times in moderate doses [27, 34]. This misunderstanding has led to critics of MDMA-assisted psychotherapy citing research showing the dangers of recreational drug use as a reason not to approve this research [41, 45, 46], which is not comparing like with like.

Due to the misunderstanding that MAPS have researched MDMA as a treatment, other researchers have considered MDMA as a “treatment for other conditions”, such as alcohol misuse [37] and social anxiety in adults with autism [47], which also needs clarification. From the MAPS work, we are not able to conclude that MDMA is a treatment, and it is not about treatment of a so-called ‘disorder’; it involves the participating individual taking MDMA to open their mind sufficiently to engage with psychotherapy and to deal with whatever problems they are experiencing. To date this appears to have been particularly helpful for people living with psychological trauma-related problems. MDMA-assisted psychotherapy is not a therapy for a ‘disorder’; it is a therapy for the person. As is inherent in a reductionist approach to understanding the complexities of human beings, it ignores the person at the heart of it, which is actually the mechanism of action studied to date in this work.

### Loss of objectivity

Research into the clinical use of illegal substances seems to often cause a worrying loss of objectivity and ability to critically appraise, fundamental to the academic process. The MAPS model suggests that two or three MDMA-assisted psychotherapy sessions may produce long-term benefits. “However, this does not fit with current models of pharmacotherapy, where regular dosing is required to maintain the altered neurotransmitter status (viz. antidepressants or antipsychotics [45, p. 300])”. As Doblin et al. [48] reply, “Parrott’s statement misses the point” (p. 106). Parrott [46] also states “…psychotherapists…suggest that the most important aspect is always the psychotherapy element and that MDMA may simply facilitate the process…it will always be far safer to undertake psychotherapy without using co-drugs…cognitive restructuring via high quality psychotherapy should always be the main element” (p. 41) and “Psychotherapy may be safer without the use of stimulant co-drugs” [45, p. 300]. It has not been suggested that MDMA-assisted psychotherapy is suitable for everyone who has had a difficult time; MDMA has thus far been found helpful in small studies in assisting psychotherapy for people who are living with the psychological consequences of trauma who are not able to manage their feelings by psychotherapy alone. Further research is required to establish the safety and efficacy of this method as a potential first-line treatment option for all diagnosed with PTSD, and Parrott appears to simultaneously miss the point and make the point.

Parrott [41] provides hypothetical examples of what might happen after MDMA-assisted psychotherapy that he states are based on evidence (although it is not clear if this evidence is of the harmful effects of regular ecstasy use or clinically administered MDMA); in one case leading to ecstasy addiction and a failed suicide attempt; in the other leading a traumatised military veteran to violently attack a stranger. Both cases then result in the individuals suing their therapists and a pharmaceutical company. While it is of course vital that any negative consequences of taking MDMA are explored, recorded and understood (as the MAPS team have done), conjecture such as this should not appear in an academic paper and Doblin et al. [48] confirm that “These surreal fabrications have never even remotely occurred in controlled trials of MDMA-assisted psychotherapy” (p.107).

In attempting to manage the legal and political minefield inherent in this type of research, it is imperative that we are precise about its exact nature. Sessa and Nutt [49] highlight the challenges of getting this type of work approved, rightly question the involvement of politics in clinical research, and draw attention to important evidence suggesting that MDMA could safely be re-classified as a Schedule 2 drug, making carrying out clinical research more straightforward. “We call on the Advisory Council on the Misuse of Drugs to recommend MDMA become a Schedule 2 drug…”; however, they go on to add “…to explore the full potential of MDMA as a medicine for treatment-resistant PTSD and other possible brain disorders” (p. 5), which is again misleading with regard to the aims of the work.

A valid point that has been raised is that the MDMA-assisted psychotherapy studies to date have been carried out solely by one group of people, in that all have been run and funded by MAPS, which has led some to question their impartiality [50]. However, this has sometimes been done using loaded and unhelpful language; for example, “…all the articles about MDMA-assisted psychotherapy were being written by the advocates themselves [46, 114]” and “The main proponents for MDMA-assisted therapy…” [45, p. 300]. The use of the terms ‘advocates’ and ‘proponents’ is unwarranted. I have not been able to find examples of this language being used to describe researchers working with other drugs; for example, are people researching the effects of antidepressants ever referred to as ‘advocates’ or ‘proponents’ of antidepressant medication? While it is certainly not healthy for any subject to be researched solely by one group of people, this cannot be a criticism levelled at MAPS themselves as they are at least advancing work in the field. Our focus needs to remain with assessing the quality of the MAPS research; we should evaluate their methodology, data analysis, interpretation of findings and conclusions drawn, and we should then conclude that more research is required to understand fully the potential, limitations and risks associated with MDMA-assisted psychotherapy [39, 51], preferably with other independent research teams involved to expand the validity and reliability of research findings. We should not resort to what feels like a dismissal of the work based on the fact that the team are ‘advocates’, criticise the use of MDMA because ecstasy has been found to be harmful or use hypothetical examples of what might happen if someone takes MDMA when it so far has not happened in the trials being referred to. “Scepticism is warranted, but cynical non-scientific bias can result in therapeutic nihilism” [52, p. 418].

## Conclusions

In summary, MDMA-assisted psychotherapy is a promising approach to helping people who have experienced psychological trauma and who have not been able to resolve their resulting problems through existing treatment options. Research into its effectiveness, safety and long-term benefits is still in its infancy, however there is already a great deal of misunderstanding of its aims and findings in the academic literature. If this is to get the research attention it deserves, it is essential that we report this accurately and objectively. MDMA may provide a bridge to effectively overcome the gap between psychotherapy and psychopharmacology, thereby facilitating the integration of an exciting new holistic approach to psychopathology [53] and “…we must not allow preconceptions, politics or puritanism…” [52, p. 420] or misinformation to get in the way of this.
